# Seed Transmission of *Epichloë* Endophytes in *Lolium perenne* Is Heavily Influenced by Host Genetics

**DOI:** 10.3389/fpls.2018.01580

**Published:** 2018-11-13

**Authors:** Milan Gagic, Marty J. Faville, Wei Zhang, Natasha T. Forester, M. Philip Rolston, Richard D. Johnson, Siva Ganesh, John P. Koolaard, H. Sydney Easton, Debbie Hudson, Linda J. Johnson, Christina D. Moon, Christine R. Voisey

**Affiliations:** ^1^AgResearch, Grasslands Research Centre, Palmerston North, New Zealand; ^2^The Foundation for Arable Research, Christchurch, New Zealand

**Keywords:** endophyte transmission efficiency, environment, *Epichloë festucae* var. *lolii*, genotyping-by-sequencing, perennial ryegrass, symbiosis, plant–microbe interactions

## Abstract

Vertical transmission of symbiotic *Epichloë* endophytes from host grasses into progeny seed is the primary mechanism by which the next generation of plants is colonized. This process is often imperfect, resulting in endophyte-free seedlings which may have poor ecological fitness if the endophyte confers protective benefits to its host. In this study, we investigated the influence of host genetics and environment on the vertical transmission of *Epichloë festucae* var. *lolii* strain AR37 in the temperate forage grass *Lolium perenne*. The efficiency of AR37 transmission into the seed of over 500 plant genotypes from five genetically diverse breeding populations was determined. In Populations I–III, which had undergone previous selection for high seed infection by AR37, mean transmission was 88, 93, and 92%, respectively. However, in Populations IV and V, which had not undergone previous selection, mean transmission was 69 and 70%, respectively. The transmission values, together with single-nucleotide polymorphism data obtained using genotyping-by-sequencing for each host, was used to develop a genomic prediction model for AR37 seed transmission. The predictive ability of the model was estimated at *r* = 0.54. While host genotype contributed greatly to differences in AR37 seed transmission, undefined environmental variables also contributed significantly to seed transmission across different years and geographic locations. There was evidence for a small host genotype-by-environment effect; however this was less pronounced than genotype or environment alone. Analysis of endophyte infection levels in parent plants within Populations I and IV revealed a loss of endophyte infection over time in Population IV only. This population also had lower average tiller infection frequencies than Population I, suggesting that AR37 failed to colonize all the daughter tillers and therefore seeds. However, we also observed that infection of seed by AR37 may fail during or after initiation of floral development from plants where all tillers remained endophyte-infected over time. While the effects of environment and host genotype on fungal endophyte transmission have been evaluated previously, this is the first study that quantifies the relative impacts of host genetics and environment on endophyte vertical transmission.

## Introduction

Grasslands account for one quarter of the world’s vegetation (Jones and Lazenby, 1988), and in temperate areas, ryegrass (*Lolium perenne*, family Poaceae) is one of the most widely used cool-season species in pastoral agriculture. Many members of the subfamily, Poöideae, have co-evolved with symbiotic fungi of the genus *Epichloë* (Leuchtmann et al., 2014) (family Clavicipitaceae), biotrophic endophytes that primarily occupy the intercellular spaces of the aerial parts of the plant. *Epichloë* symbionts are typically systemic throughout aerial grass tissues and persist throughout the life of the host where they can confer a variety of abiotic and biotic benefits (Schardl et al., 2004). Transmission of *Epichloë* endophytes between plants may occur through the horizontal spread of sexually derived fungal ascospores produced on ectopic hyphal stromata (Bultman et al., 1995; Chung and Schardl, 1997; Brem and Leuchtmann, 1999). However, in asymptomatic associations, the endophytes are transmitted vertically and asexually, where they colonize the florets of the host, including the ovaries, and are transmitted to the developing embryo (Sampson, 1933; Philipson and Christey, 1986; Liu et al., 2017; Zhang et al., 2017). Depending on the species of the host and endophyte, *Epichloë* transmission strategies may be entirely vertical or horizontal, or both strategies may take place simultaneously on the same plant (White, 1988; Tadych et al., 2014).

In grasslands with intense insect pest pressure, such as the temperate pastures of New Zealand, Australia, and the Americas, transmission of viable *Epichloë* hyphae to the seed is important for optimal persistence of newly sown pastures (Hill et al., 2005). The fungi synthesize bioactive alkaloids and other metabolites (Tanaka et al., 2012; Johnson et al., 2013a) that protect plants against biotic and abiotic stresses (Prestidge and Gallagher, 1988; Arachevaleta et al., 1989; Siegel et al., 1990). However, since some classes of bioactives are also toxic to vertebrates, the productivity and health of grazing livestock may be severely impacted (Bacon et al., 1977; Fletcher and Harvey, 1981). Asymptomatic proprietary endophyte strains with alkaloid profiles that protect host plants against many insects and minimize adverse animal health effects, are in widespread use in pastoral agriculture, particularly in New Zealand (Johnson et al., 2013a; Young et al., 2013). However, further strains with bioactivity against significant insect pests continue to be sought (Johnson et al., 2013a). Endophyte strains with desirable characteristics can be readily introduced into the seedlings of commercial grass cultivars, thus combining the best characteristics of the host and the endophyte in novel symbiotic associations that are made commercially available to farmers in forage seed (Johnson et al., 2013a).

Efficient vertical transmission of endophyte hyphae into the seed is therefore an important prerequisite for agricultural utilization of novel *Epichloë* strains. However, this process is often imperfect in both naturally occurring and novel endophyte–grass associations (Afkhami and Rudgers, 2008; Gundel et al., 2009; Rudgers et al., 2010; Gibert and Hazard, 2013). From an ecological perspective, imperfect transmission allows the grass to sanction against the endophyte to reduce costs when resources are limiting (Gundel et al., 2011a), or when the incidence of abiotic and biotic stresses are low and endophyte-free progeny have the same or greater fitness than endophyte-infected plants. While endophyte infection rates in natural grasslands has been studied widely (for examples, see Siegel et al., 1984; Shelby and Dalrymple, 1987; Leyronas and Raynal, 2001), much of the published research is descriptive, and there are few reports on the genetic or physiological mechanisms involved. Similarly, few studies have examined the influence of genetic compatibility between the host and endophyte on vertical transmission frequency, and these have yielded varied observations (Gundel et al., 2010, 2012; Saikkonen et al., 2010; Noh and Ju, 2012). While several studies have reported effects of genetic compatibility between host and endophyte on transmission frequency (Gundel et al., 2010; Saikkonen et al., 2010; Noh and Ju, 2012), in a study that examined *E. occultans* transmission in a range of *L. multiflorum* genetic backgrounds, host genetic background did not appear to strongly influence transmission (Gundel et al., 2012).

The advent of high density single-nucleotide polymorphism (SNP) marker platforms, such as genotyping-by-sequencing (GBS) (Elshire et al., 2011) offers the opportunity to further investigate the extent that host genetic background plays in endophyte vertical transmission. GBS provides a tool for enabling genomic selection (GS; Meuwissen et al., 2001), where the SNPs are used directly to predict the breeding values of individuals, rather than traditional selection strategies that require potentially time-consuming phenotypic measurements between each cycle of selection. In addition, the identification of informative SNP markers can be used to provide a platform to identify discrete host genetic factors underlying the trait of interest. In contrast to marker-assisted selection, the goal of GS is not to identify and leverage the effects of individual markers, but is to capture as much of the genetic variation for a trait as possible as a breeding value. SNP marker effects on a trait are first estimated in a training set of individuals, sampled from a breeding program, for which there are both genome-wide SNP genotype and trait phenotype data available. These data are used to derive a statistical model that integrates the effects of all of the SNP markers simultaneously, for prediction of the phenotype. The model can then be used to predict the phenotype of individuals within a selection population that have been genotyped only. This predicted phenotype is referred to as a genomic-estimated breeding value (GEBV). The predictive ability (PA) of the model, defined as the correlation between observed phenotype and the GEBV, is used to evaluate the effectiveness of the model. In forages, such as perennial ryegrass, application of GS is expected to increase the rate of genetic gain for a trait by reducing the length of the selection cycle (Heffner et al., 2010), as well as by enabling within-family selection pressure to be applied (Casler and Brummer, 2008; Faville et al., 2018). Recently, GBS was applied to perennial ryegrass to generate genomic prediction models for multi-year dry matter yield, and demonstrated the potential for genetic gain improvement in this trait (Faville et al., 2018). Use of this approach to breed for improved endophyte transmission is feasible, and the identification of SNPs that segregate with endophyte transmission rates may also provide new leads into the underlying genes and mechanisms.

Environmental variables are also thought to alter endophyte seed transmission efficiency (Gundel et al., 2011b), though direct evidence for this is also sparse (Gundel et al., 2011a, 2012; Noh and Ju, 2012; Parisi et al., 2012; Wang et al., 2018). Based on their impacts on plant growth, endophyte density, and predicted costs and benefits of maintaining the endophyte symbiosis, environmental factors thought to impact vertical transmission include resource availability, environmental cues, abiotic stresses, and biotic stresses (Gundel et al., 2011b). However, the degree to which these factors affect vertical transmission, and thus the likely underpinning mechanisms, remain poorly understood (Gundel et al., 2011b, 2012).

**FIGURE 1:**
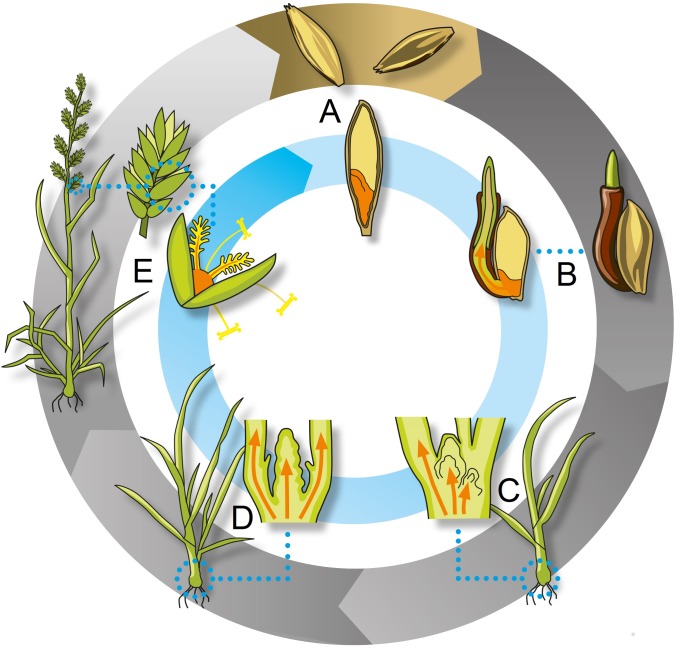
Vertical transmission of *Epichloë festucae* var. *lolii* hyphae. The outer ring represents the grass reproductive cycle (both vegetative and sexual), and the inner ring the fungal vertical transmission cycle (asexual only). Within mature seeds **(A)**, hyphae (orange) are present in the embryo, as well as a number of other seed structures such as the scutellum, and a region between the embryo and the endosperm called the “infection layer” (Philipson and Christey, 1986; Card et al., 2011). Upon germination **(B)**, hyphae within the embryo colonize the seedling, including the shoot apical meristem, and developing shoot organ primordia using hyphal tip growth that allows the branching of hyphae required to colonize and ramify through meristem and organ primordia. By contrast, hyphal extension in growing leaves occurs primarily through intercalary compartment growth until the leaf matures (Christensen et al., 2008). Each leaf subtends an axillary bud which is colonized prior to tiller outgrowth, thus enabling the hyphae to colonize the shoot apex of the newly formed daughter tillers **(C)** and from there, all nascent aerial plant structures. The resulting grass plants are comprised of a hierarchy of tillers and it is not uncommon for all tillers of an infected grass plant to contain hyphae. However, organ primordia that have escaped hyphal colonization are generally observed to develop endophyte free (Wilson and Easton, 1997; Christensen et al., 2000). Under environmental cues (temperature, light duration), each vegetative shoot apex has the potential to differentiate into a reproductive inflorescence primordium **(D)**. Hyphae colonize the developing reproductive tissues, and can be identified among the cells of most floral tissues, including the ovaries **(E)**. Upon fertilization, hyphae in the ovary penetrate the embryo and are incorporated into other seed structures (Sampson, 1933; Liu et al., 2017; Zhang et al., 2017). It is noted that hyphae have not been observed in grass pollen (Liu et al., 2017) and thus the transmission of endophytes into seed through the asexual cycle is generally deemed to be exclusively maternal. Moreover, there are examples where epibiotic conidia have been shown to be capable of horizontal spread (Tadych et al., 2012, 2014). However, the extent to which this may occur is unclear, and has not been documented in AR37 associations with *L. perenne*.

Successful vertical transmission of *Epichloë* endophyte into the seed is dependent on the coordinated growth and development of both the grass and endophyte (see Figure 1 for detailed description). Ultimately, the capacity of hyphae to colonize host meristematic tissues that will later become floral structures and seed is critical. In the context of the host life cycle therefore, three key developmental stages are recognized at which vertical transmission of *Epichloë* into progeny plants *via* the seed may fail (Figure 1). First, seedlings may become endophyte free after germination of infected seeds if hyphae fail to colonize the shoot apex of the developing seedling. Second, hyphae in the shoot apex may fail to colonize the axillary meristems that will form daughter tillers, resulting in endophyte-free tillers. Third, hyphae in the inflorescence primordium may not adequately colonize the developing florets, ovaries, and seeds, which may then contain little or no hyphae.

Seed transmission of *E. festucae* var. *lolii* strain AR37, a commercially available ryegrass endophyte that confers broad spectrum insect pest resistance (Popay and Wyatt, 1995; Popay and Thom, 2009; Johnson et al., 2013a), can be too low to achieve quality control specifications for seed production or for sowing pastures. This issue is highly costly for the seed industry, and can result in the disposal of seed before point of sale. There is therefore significant interest in understanding the main determinants of vertical transmission in the host–endophyte interaction in order to enhance the endophyte infection rates of seed, and hence agronomic benefit to farmers.

This study is part of a wider investigation that aims to understand the main factors and mechanisms that underlie high and low vertical transmission rates of endophytes in host grasses, in order to develop strategies that improve endophyte transmission into commercial *L. perenne* seed. In this paper, we primarily explore the influence of host genetics on endophyte vertical transmission. By using GBS information from five genetically diverse *L. perenne* populations (Faville et al., 2018) infected with *E. festucae* var. *lolii* AR37, we examine host genetic and environmental influences on the efficiency of vertical transmission in over 500 plant genotypes. These data are used to determine the potential for using GS as a means to increase the rate of genetic gain for AR37 transmission *via* plant breeding. The host genotype data will also provide a foundation for future studies to gain insights into genes, biochemical pathways, and biological processes that are associated with mechanisms of endophyte transmission. In addition, further phenotypic characterization of the host plants (e.g., endophyte biomass, tiller infection rates) within the populations is used to examine developmental stages of the host–endophyte life cycle to gain insight into critical processes that may contribute to enhancing vertical transmission rates in commercial *L. perenne* seed.

## Materials and Methods

### Plant Populations

All observations in this study were based on individual plants from a GS training set consisting of five *L. perenne* breeding populations (Pops I–V) infected with *E. festucae* var. *lolii* strain AR37, previously described by Faville et al. (2018). These populations were originally established to enable development of genomic prediction models for agronomic traits *via* GBS. The parent plants within each population were randomly selected from the crosses described in Table 1, apart from Population III, which has a defined family structure (i.e., was comprised of families of related half-siblings, individuals which share a single maternal parent but may have different paternal parents) as described in Supplementary Table S1. Populations I–III had undergone one cycle of phenotypic selection for AR37 seed transmission, whereas Populations IV and V had not. For the current study, 577 AR37-infected plant genotypes were used (Table 1).

**Table 1:** Description of *L. perenne* populations used in this study.

Population	Description^1^	Number of parent plants^3^	Genetic relatedness^4^
Pop I	Cross between two late-flowering New Zealand cultivars. Population has undergone one cycle of selection for endophyte transmission.	112 (97)	0.33
Pop II	Mid-flowering New Zealand cultivar × Spanish ecotypes. Population has undergone one cycle of selection for endophyte transmission.	113 (110)	0.32
Pop III	Cross among five late-flowering New Zealand cultivars.^2^ Population has undergone one cycle of selection for endophyte transmission.	121 (113)	0.27
Pop IV	Late-flowering New Zealand cultivar × European cultivar.	113 (95)	0.43
Pop V	Two New Zealand cultivars × Spanish ecotype.	118 (109)	0.34

*^1^The populations have been previously described in Faville et al. (2018). ^*2*^The genetic structure of Population III is described in Supplementary Table S1. ^3^Total number of parent plants within each population, with number for which % viable endophyte transmission was determined in 2012/13 is shown in brackets. ^4^Mean within-population genetic relatedness coefficient, derived from a KGD genomic relationship matrix based on 1.02 M SNPs.*

Plants comprising the five populations were raised from seed in 2012, and were then grown in 2 L pots filled with standard seed raising mix (screened fine bark 75% [w/w], coir fiber 12.5% [w/w], pumice 12.5% [w/w], and Osmocote [Scotts, Bella Vista, NSW, Australia] 0.5% [w/w]). Plants were divided into two ramets of 6–10 tillers and re-potted as part of general maintenance as they outgrew their pots, or as needed for studies that required plant replicates. Plants were grown at the Grasslands Research Centre (GRLDS, 40°22′53.2″S 175°36′42.5″E) in Palmerston North, Manawatu, New Zealand.

### Seed Production in Populations I–V (2012/2013)

Plants within Populations I–V were intercrossed in 3 m × 3 m isolation glasshouses in the Southern Hemisphere spring of 2012. Each of the five polycrosses included all of the plants from only one population, with no pollen flow between populations, and each plant genotype was represented by two clonal copies. The two clones were spatially separated in the isolation glasshouse to facilitate exposure to pollen from as many donors as possible. Air was internally circulated within the glasshouse to distribute pollen shed from the plants throughout the space. The progeny from each maternal parent plant were half-siblings that shared the same maternal line, though with a number of different paternal contributors (pollinators). Seeds from individual maternal parent plants were harvested in the summer of 2013, as soon as the start of seed drop was detected. Late-maturing plants were allowed an additional week before the harvest. Seeds from the two clones of each maternal parent were pooled, producing up to 118 maternal half-sibling families per population. Seed was cleaned to achieve a weight of approximately 1.8 g per 1000 seed, and immediately stored at 30 ± 5% relative humidity and 0 ± 5°C at the Margot Forde Germplasm Centre (Palmerston North, New Zealand) until required.

### Seed Production in Populations I and IV (2013/2014)

In 2013/14, the seed transmission assessment was repeated outdoors for a subset of individuals from breeding populations Population I (*n* = 74) and Population IV (*n* = 109) only, but at two locations: one in the North Island (GRLDS) and one in the South Island at the Lincoln Research Centre (LINC, 43°38′34.2″S 172°28′24.2″E) in Lincoln, Canterbury, New Zealand. In September 2013, plants that were grown in pots at GRLDS were divided into two to four clones. One clone was re-potted in GRLDS, whereas the second clone was transported to LINC as a bare-rooted ramet and transplanted directly into the soil. For this trial, two clonal replicates were also used for 14–22 of the plants in each population at each location. Climate data for GRLDS and LINC during 2013/2014 were obtained from the New Zealand National Climate Database^1^ (CLIDB) from the observatories at AgResearch Grasslands (40°22′55.2″S 175°36′32.4″E) and Plant & Food Research, Lincoln (43°37′33.6″S 175°28′12″E) (Supplementary Figure S1). Temperature information for GRLDS in January and February of 2014, as well as the rainfall information for GRLDS in February of 2014 were obtained from a close observatory (40°19′6.9″S 175°36′52.5″E) due to data missing in the observatory at AgResearch Grasslands (Supplementary Figure S1).

### Measurement of Endophyte Transmission From Maternal Plant to Seedlings

The standard assessment method for vertical transmission of viable endophyte to the progeny is *via* the detection of endophyte in the pseudostems of young seedlings (Rolston and Agee, 2007), and hereafter is referred to as “% viable endophyte transmission.” To measure % viable endophyte transmission levels from maternal parent plants to seedlings, approximately 120 seeds (0.2 g) from each maternal parent seed pool was divided into three samples (termed “sample” for the statistical analysis described in Section “Statistical Analyses”). Each sample (∼0.067 g) was randomly allocated to plastic trays of 12 × 12 cells, with samples from three parent plants individually sown in blocks within each tray. After planting, the seeds were placed into a glasshouse nursery under natural day-length for 7 days to germinate, and then transferred outdoors to grow for a further 6 weeks. The number of seeds that had germinated and developed into seedlings in this time was recorded. One tiller from each plant was cut at approximately 5 mm from soil level. The freshly cut end of the tiller was blotted onto a nitrocellulose membrane. Endophyte detection *via* tissue-print immunoassay was performed using a modification of the method by Hahn et al. (2003) described by Simpson et al. (2012) using polyclonal rabbit antibodies that had been raised against homogenized mycelium of *E. festucae* var. *lolii* strain Lp5.

This procedure was used to assess the seed collected in the summer of 2012 from Populations I to V grown at GRLDS, using a randomized sowing schedule over five intervals (from 23/9/2013 until 25/11/2013) the following spring. Approximately 12,000 seedlings were assayed per population. The % viable endophyte transmission rates were similarly determined for seed collected from the Populations I and IV maternal parent plants grown in GRLDS and LINC in 2013/14.

### Detection of Endophyte in Seed by High-Resolution Melting Analysis

While we have used the standard assessment for seed transmission *via* detection of viable endophyte in young seedlings, it is possible that seed was colonized, but the endophyte did not establish within the seedling, and thus was not detected in seedlings. As such, we sought to analyze maternal parent genotypes, across a range of observed % viable endophyte transmission values, for seed colonization. The maternal plants from all five populations from the GRLDS 2013 seed harvest were ranked in descending order according to their endophyte transmission values, as established through the immunoassay of seedlings. From each population, 9–20 individual maternal plants were selected to obtain a range of % viable endophyte transmission values at each decile. Individual seeds (*n* = 24) from the selected maternal plants were tested for the presence of AR37 DNA at a commercial facility using a PCR method with high resolution melt analysis performed by Slipstream Automation Ltd. (Palmerston North, New Zealand) (Johnson and Voisey, 2017). Briefly, high throughput DNA extraction was used to isolate DNA from individual seeds. A pair of primers designed to PCR amplify a ribosomal repeat intergenic spacer fragment of AR37 was used to assay DNA samples on a Light Cycler 480 (Roche, Indianapolis). Scoring of samples was according to the high resolution melting dissociation curve obtained in response to increasing temperature, and enabled both the presence and identity of the endophyte strain to be confirmed (Johnson and Voisey, 2017).

### Genotyping-by-Sequencing and Genomic Prediction Modeling

DNA isolation and GBS of 577 parent plants formed a training population that was described in Faville et al. (2018). Of these, only 524 individuals with both genotypic and phenotypic information available were used in the present study. Briefly, DNA was isolated from leaf material using the method of Whitlock et al. (2008), with some modifications. GBS libraries were generated following the methodology of Elshire et al. (2011), with DNA digested using *Ape*KI (New England Biolabs, Ipswich, MA, United States). Each library was passed through a Pippin Prep^TM^ DNA size selector (Sage Science, Beverly, MA, United States) to isolate fragments between 193 and 313 bp, and were then sequenced on two lanes of an Illumina HiSeq 2500 (Illumina, San Diego, CA, United States) at AgResearch Invermay, New Zealand. Raw reads underwent de-multiplexing, tag alignment and SNP calling using the TASSEL 5.0 GBS pipeline (Glaubitz et al., 2014), with GBS tags aligned to a perennial ryegrass reference genome (Byrne et al., 2015) using Bowtie2 (Langmead and Salzberg, 2012). SNP calling was conducted jointly for all libraries, combining data for Populations I–V into a single analysis. After filtering for missing data per site (50%), minor allele frequency (>0.05) and Hardy–Weinberg disequilibrium (greater than -0.05) (Dodds et al., 2015), a total of 1,023,011 SNPs, with a mean read depth of 2.94, were obtained.

Determination of GEBVs (predicted phenotype values) for individuals in the five populations was undertaken for the 2012/2013 endophyte transmission data using four different statistical methods, as described in Faville et al. (2018): ridge regression (RR), random forest regression (RF), and genomic best linear unbiased prediction (GBLUP) using either a standard genomic relationship matrix (GBLUP) or a recently developed relatedness estimation method for low depth sequencing data (KGD-GBLUP) (Dodds et al., 2015). For all methods, except for KGD-GBLUP (no imputation), imputation of missing values was by mean imputation as described by Rutkoski et al. (2013). Predictive ability provides information on the effectiveness of the genomic prediction model. Here, PA was estimated by 10-fold cross-validation. To do this, the full set of training population individuals was split into training (90% of individuals) and validation (10% of individuals) groups. A predictive model was derived using data from the training group only. The model was used to predict GEBVs for individuals in the validation group, using their genotype data. This was repeated five times for each of the models (GBLUP, KGD-BLUP, RR, and RF). Predictive ability was the mean Pearson correlation coefficient between the GEBVs predicted by the model, and the observed phenotypic values for the validation group.

### Detection of Endophyte in Tillers of Mature Plants

One of the requirements for *Epichloë* transmission into the seed is the successful infection of new daughter grass tillers, which itself is dependent on endophyte colonization of axillary buds at the base of the plant. We thus sought to determine whether the infection rate of vegetative tillers generally correlated with the endophyte transmission rate. To test this, a subset of plants from Populations I and IV were analyzed by tissue print-immunoassay (Hahn et al., 2003; Simpson et al., 2012) for endophyte presence in the tillers in 2016. At least 16 randomly selected tillers per plant were tested, and tiller infection rates were compared to AR37 transmission rates in seed harvested in Jan 2013 from the respective maternal genotypes.

### Laser Scanning Confocal Microscopy of AR37 in Vegetative Tiller Crowns

To observe the distribution of endophyte hyphae within the tiller crown (TC) regions of maternal parent plants, laser scanning confocal microscopy was used. Tissue samples were prepared from vegetative tillers that were randomly selected from maternal plants. Tiller segments (from the root-shoot junction to approximately 3 cm above), were embedded in 6% (w/v) agarose (Invitrogen, Carlsbad CA, United States) in water. Once set, the pseudostems were trimmed to 1 cm above the root–shoot junction. These tissues included meristematic, expanding and expanded plant cells of leaves, stems, and primordia, where the morphology of the shoot apical meristem can be used as a guide to determine the developmental age of the tiller (Jeater, 1956). The tissues were hand-sectioned longitudinally using a razor blade (ProSciTech, Brisbane, QLD, Australia). Hyphae were stained with aniline blue diammonium salt (Sigma Chemicals Co., Milan, Italy) and Alexa Fluor-488 WGA (Molecular Probes, Eugene, OR, United States) as described (Becker et al., 2015). Specimens were observed under an inverted confocal laser scanning microscope (FV10i-w; Olympus, Tokyo, Japan) with an UPLSAPO-equivalent phase contrast 10× objective using FV10i-ASW 3.1 Viewer software (Olympus, Center Valley, PA, United States). Excitation of aniline blue was at 405 nm with detection using a 420–460 nm barrier filter (setting “Blue”). Excitation of Alexa Fluor 488 was at 473 nm with detection using a 490–590 nm barrier filter setting. A white pseudo-color was assigned to the emission fluorescence from the Alexa Fluor 488-stained structures.

### Quantification of Endophyte Biomass in Vegetative Tiller Crowns

Quantitative real-time PCR (qPCR) was used to measure endophyte biomass in vegetative TCs. The concentration of AR37 DNA in vegetative TCs, including the basal 1 cm of the pseudostem, was determined by qPCR based on a 153 bp DNA fragment amplified from the AR37 non-ribosomal peptide synthetase gene, NRPS1, as previously described (Rasmussen et al., 2007). Tiller segments (cut from the root–shoot junction to 1 cm above) were tissue printed onto nitrocellulose membrane, frozen immediately in liquid nitrogen, and stored at –20°C until required. The endophyte infection status of the cut tillers was confirmed by tissue-print immunoassay (as described earlier). Total DNA was prepared from infected plant tissues, and also from an axenic mycelial culture of AR37 (to generate an AR37 standard curve for qPCR). Tissues were ground to a powder in liquid nitrogen. Plant DNA was extracted using a Geneaid^®^ Genomic DNA Mini kit (Geneaid Biotech Ltd., Taipei, Taiwan), and fungal DNA extracted using the ZR Fungal/Bacterial DNA MiniPrep kit (Zymo, Orange, CA, United States) according to manufacturers’ instructions. DNA was quantified using a Qubit Fluorometer with the Quant-iT^TM^ dsDNA HS Assay kit (Invitrogen). Quantitative PCR amplification was performed using KAPA SYBR^®^ FAST qPCR reagents (KAPA Biosystems, Boston, MA, United States) in a 10-μL reaction containing 50 ng DNA and 200 nM each of forward primer 5′-GTCCGATCATTCCAAGCTCGTT-3′, and reverse primer 5′-TGGTGGGAAGTTCCCTGCAG-3′ in a Light-Cycler 480 (Roche Applied Science, Mannheim, Germany). Quantitative PCR was performed in triplicate for each biological sample and each dilution of the standard curve. The standard curve was prepared from AR37 genomic DNA at 15, 7.5, 0.75, 0.075, 0.0075, and 0.00075 ng per reaction using the Light-Cycler 480 associated software. Endophyte biomass is expressed as pg of the NRPS1 gene per ng of total genomic DNA.

### Statistical Analyses

All standard statistical analyses were conducted using GenStat (VSN International, 2015) and R software version 3.4.3 (R Core Team, 2017). The presence or absence of endophyte within progeny plants is a binary variable, and hence the proportion of endophyte-infected progeny from a parental genotype was assessed using binomial tests. Endophyte infection % among maternal plants within populations did not satisfy assumptions of normality, thus non-parametric methods such as Kruskal–Wallis one-way analysis of variance, Dunn’s multiple comparison, and Wilcoxon rank-sum tests were used to compare populations. Within Population III, which had a known family structure (Supplementary Table S1), analysis of variance (ANOVA) of arcsin transformed data was used to assess for variation in % viable endophyte transmission among the half-sibling families. The concordance of variables among genotypes within populations was assessed using the Spearman rank correlation test. Relationships between variables were investigated by fitting simple linear regression models.

For the AR37 seed transmission trial repeated at two sites in 2013/14, using plants from Population I and Population IV, data were also analyzed using the linear mixed models option in GenStat. The two populations were analyzed separately. In each case a random linear mixed model was applied, using the REML algorithm, with genotype, sample and replicate effects considered as random and site as a fixed effect. The linear model also included a genotype-by-site interaction effect. The significance of an estimated variance component was determined by the ratio of the component relative to its standard error. If the variance component of a model term was more than two standard errors from zero, then the variance component was considered significant (*P* < 0.05). The variance components generated from REML analysis were used to estimate clonal repeatability (*R_c_*). This value represents the proportion of the phenotypic variance (phenotypic differences among individuals) observed in the population that is attributable to genotypic variance (genetic differences among individuals). As such, it represents an upper limit for the degree of genetic determination for the trait (Falconer, 1989):

$$R_{c}=\frac{\sigma_{g}^{2}}{\sigma_{g}^{2}+\frac{\sigma_{gl}^{2}}{nl}+\left(\frac{\sigma_{g}^{2}}{n_{r}n_{l}}\right)}$$

where $\sigma_{g}^{2}$ = genotypic component of variance, $\sigma_{\varepsilon }^{2}$ = residual variance of genotypes, $\sigma_{gl}^{2}$ = genotype-by-site component of variance, *n*_*r*_ = number of replications, and *n*_*l*_ = number of locations.

## Results

### Vertical Transmission of *E. festucae* var. *lolii* AR37 to *L. perenne* Progeny

**FIGURE 2:**
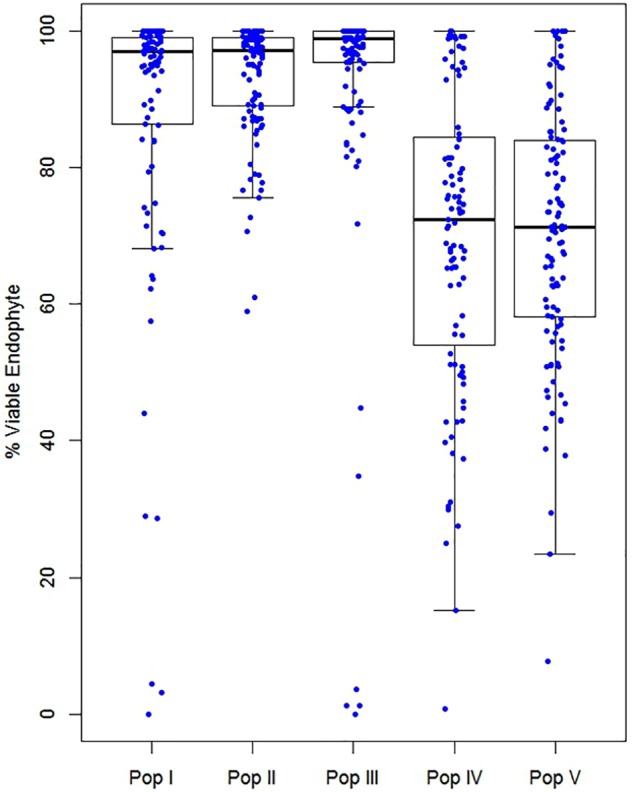
Box plots of AR37 transmission into seedlings in Populations I–V. Seeds were harvested from parental lines grown at the Grasslands Research Centre (GRLDS) in 2013, and emergent seedlings were tested for the presence of viable endophyte by tissue-print immunoassay.

Assessment of the % viable endophyte transmission rate for Populations I–V from seeds harvested in 2013 are shown in Figure 2. Populations I–III, which previously had undergone one cycle of phenotypic selection for AR37 transmission in seed, the maternal lines averaged 88, 93, and 92% transmission, respectively. By contrast, Populations IV and V averaged 69 and 70% transmission, respectively, implying successful selection and a strong host genetic effect on AR37 vertical transmission. No significant difference was observed for the mean transmission rate for each population over the different sowing dates. However, significant differences were detected among the populations with respect to the distribution of endophyte transmission rates (Kruskal–Wallis analysis of variance: 4df, chi-squared = 511.78, *P* < 0.001). *Post hoc* analyses between pairs of ryegrass populations using Dunn’s test revealed significant differences for all pairs of populations (*P* < 0.05) except between Populations IV and V (*P* = 0.23).

### Host Genetic Influence on AR37 Vertical Transmission Within Population III

Since Population III is comprised of families of genetically related individuals (Supplementary Table S1), we used ANOVA to determine whether the mean AR37% viable endophyte transmission values differed between families. Analysis of these data (Supplementary Table S2) showed that the variation among half-sibling families for endophyte transmission is significant (49, 63 df, *F* = 2.04, *P* = 0.021), indicating additive genetic variation. This result demonstrated that the half-sibling family (additive genetic) effect is small (narrow-sense heritability barely 1% for this experiment) but does exist, and helps to explain variation in endophyte transmission values.

### Genomic Prediction Models of AR37 Vertical Transmission Using GBS SNP Data

**FIGURE 3:**
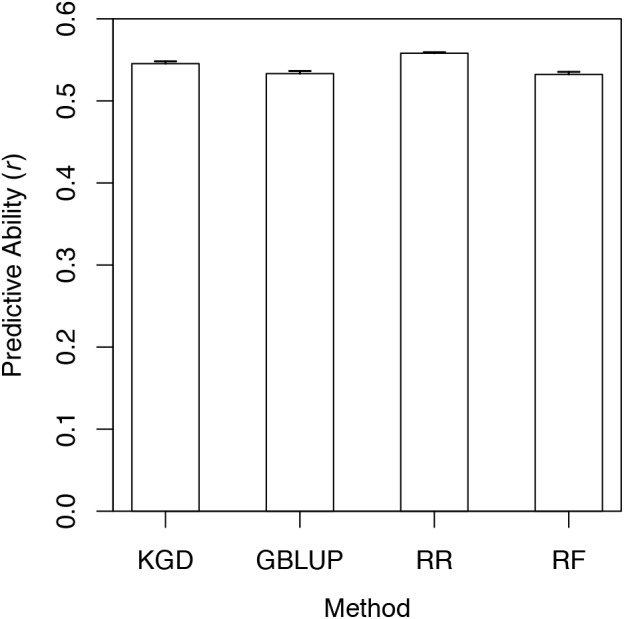
Mean (*n* = 5) 10-fold cross-validation predictive ability in a perennial ryegrass training set for % viable endophyte transmission, determined using four statistical models and assessed as Pearson correlation between observed phenotype (BLUP-adjusted mean) and GEBV. The RR model used 249,546 SNPs (largest SNP dataset able to be dealt with computationally by this method) while GBLUP, KGD, and RF used 1,023,011 SNPs. Error bars are standard errors.

Genomic prediction models based on four different statistical approaches were assessed for PA (*r*) for % viable endophyte transmission, for individuals randomly sampled from the full training set. For KGD (Supplementary Table S3), GBLUP, and RF, 1,023,011 SNPs were used in the models. Due to computational limitations, only a smaller set of 249,546 SNPs was able to be used for RR. Across all four statistical methods, mean *r* for % viable endophyte transmission was 0.54. There were no significant differences (*P* < 0.05) in PA among the four statistical methods (Figure 3). Because the phenotypic dataset was considerably left-skewed, *r* was also estimated from a reduced dataset with similar numbers of individuals within each of five phenotypic bins, and was found to be the same as that estimated for the full dataset (Supplementary Figure S2).

### The Impact of Environmental Variation on AR37 Vertical Transmission

To evaluate the role of environment on endophyte transmission into seed, we tested for AR37 vertical transmission in genotype clones grown in two geographic locations (GRLDS and LINC) that differed both climatically and geologically; and over consecutive years at GRLDS, where climate differences were observed (Supplementary Figure S1). Unexpectedly, a considerable proportion (∼27%) of genotypes in Population IV produced seedling progeny with no detectable endophyte in 2014 (from either GRLDS or LINC), suggesting that the maternal plants had lost endophyte infection in the previous year, and prior to floral initiation. This is in contrast to Population I, where only ∼3–4% of parent genotypes had lost endophyte infection.

**FIGURE 4:**
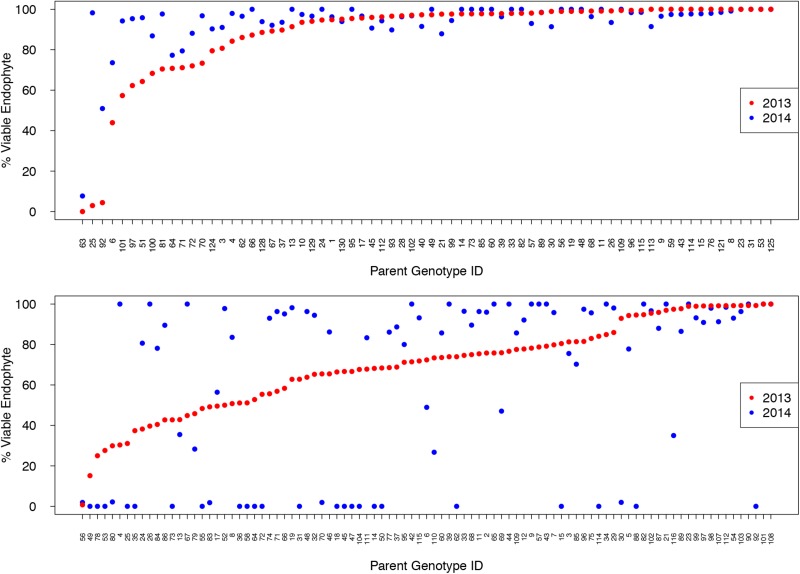
The % viable endophyte transmission for Populations I (upper panel) and IV (lower) in 2013 and 2014, with genotypes ranked from lowest to highest based on 2013 data.

Comparison of endophyte transmission rates in seeds from maternal plants grown in GRLDS in 2013 and 2014 (Figure 4) showed that, for Population I, the genotype ranks differed significantly to each other between years (Wilcoxon signed rank test with continuity correction *P* = 0.0049), while for Population IV, they did not (*P* = 0.24). AR37 transmission values at the two time points did differ for both populations however [Paired *t*-test: *t* = –3.185, *df* = 69, *P* = 0.0022 (Pop I); *t* = 2.006, df = 92, *P* = 0.048 (Pop IV)]. Moreover, for Population IV, plants with lower transmission in 2013 (e.g., <70%) were more likely to produce uninfected progeny in the following year (Figure 4).

**FIGURE 5:**
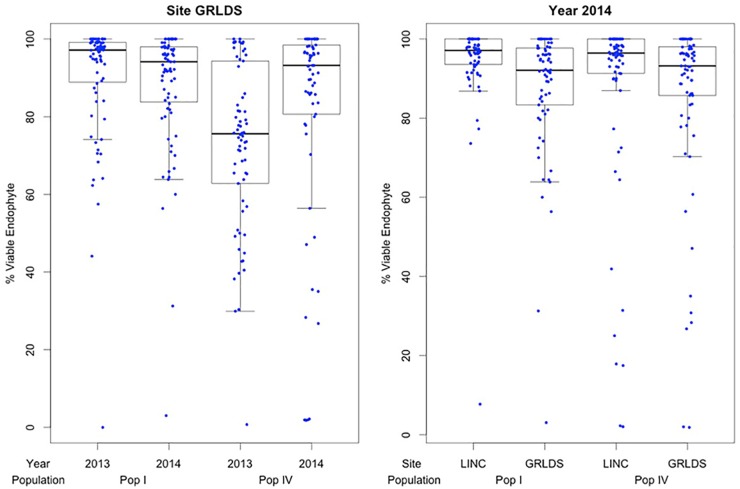
Effects of year and location on AR37 vertical transmission into *L. perenne* seed. Graphs depict comparisons of AR37 transmission for parental plant genotypes within Populations I and IV grown at the Grasslands Research Centre (GRLDS) for seed harvested in 2013 and 2014 (left), and for AR37 transmission into seed harvested from clonal replicates grown at GRLDS and at the Lincoln Research Centre (LINC) in 2014 (right). Where endophyte infection was lost, data for that parental genotype, including the corresponding location and/or harvest date data, were omitted.

Given the losses in endophyte infection of the parent plants observed in 2014, all data from affected maternal genotypes were subsequently omitted from the following analyses (Figure 5). This was to allow direct comparison of transmission rates under different environmental conditions from cloned infected maternal plants only. Endophyte transmission differed significantly when cloned maternal parents were grown in GRLDS as compared to LINC (2014 data only; Wilcoxon signed rank test: *P* < 0.0001 Population I, and *P* = 0.023 Population IV). Furthermore, in plants grown in GRLDS, AR37 transmission rates in seeds harvested in 2013 and 2014 also differed significantly (Wilcoxon signed rank test: *P* = 0.013 Population I, and *P* < 0.01 Population IV) (Figure 5). Transmission rates were higher in 2014, which was generally warmer than 2013, with higher rainfall during the spring and a drier summer (Supplementary Figure S1).

Significant host genotypic variation for endophyte transmission in both Populations I and IV was confirmed by variance components derived from linear mixed model analysis (*P* < 0.05; Table 2), and relatively low residual variance. The analysis also confirmed a significant (*P* < 0.05) effect of environment (GRLDS, LINC), as well as a small but significant (*P* < 0.05) genotype-by-environment interaction, and relatively low residual variance. Repeatability, *R_c_*, estimated for AR37 transmission in the two populations in this experiment, was consequently high at 0.82 and 0.92, respectively (Table 2). Although these values should be interpreted cautiously, due to minimal clonal replication in the datasets and imbalance in genotypes represented at both sites, this indicates a high degree of genetic determinism exists for observed phenotypic variation in endophyte transmission, in both Populations I and IV.

**Table 2:** Variance components and clonal repeatability (R_c_) estimates for endophyte transmission in Populations I and IV.

Component^1^	Pop I	Pop IV
$\sigma_{g}^{2}$	0.0177 (0.0033)	0.0269 (0.0049)
$\sigma_{gl}^{2}$	0.0030 (0.0011)	0.0048 (0.0011)
$\sigma_{\varepsilon }^{2}$	0.0106 (0.0008)	0.0055 (0.0009)
*R_c_*	0.82	0.92

*^1^Variance components (standard errors in brackets): $\sigma_{g}^{2}$ = genotypic; $\sigma_{gl}^{2}$ = genotype-by-site and $\sigma_{\varepsilon }^{2}$ = experimental error. If the variance component of a model term is more than two standard errors from zero, then the variance component is considered significant (*P* < 0.05). This is the case for all variance components shown here.*

### The Relationships Between % Viable Endophyte Transmission, Endophyte Presence in Seed, and Tiller Infection Levels

**FIGURE 6:**
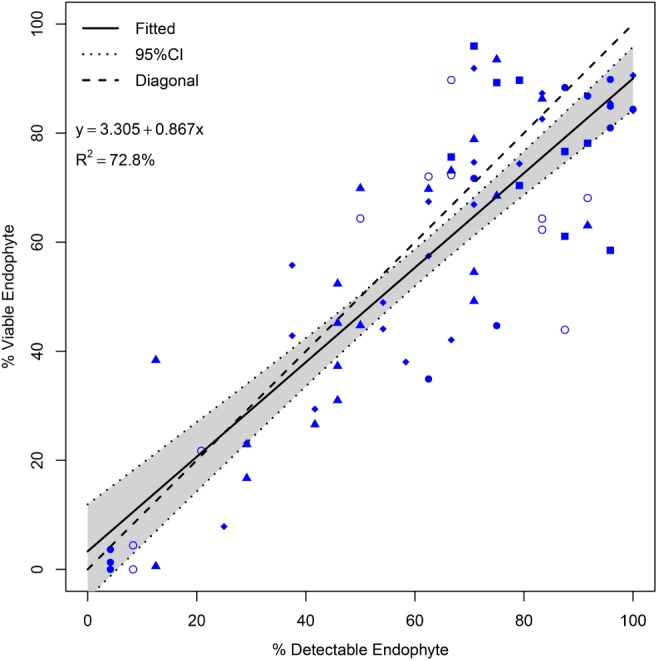
Relationship between AR37 transmission to seedlings (% viable endophyte) and presence in seed (% detectable endophyte) for seed collected at the Grasslands Research Centre (GRLDS) in 2013. Seed from selected parental genotypes within Populations I–V were tested: Pop I, empty circles (*n* = 10); Pop II, squares (*n* = 9); Pop III solid circles (*n* = 12); Pop IV, triangles (*n* = 20); and Pop V, diamonds (*n* = 20). The fitted line (solid) has an *R*^2^ = 72.8%, and the 95% confidence interval (CI) is shaded in gray.

To assess the possibility that seed was colonized by AR37, but the endophyte did not establish within the seedling (and thus was not detected in seedlings), linear regression modeling of the resulting data pairs, % viable endophyte transmission (*via* immunoassay), and endophyte presence (*via* PCR-based high-resolution melting analysis) was performed (Figure 6). This analysis indicated a strong correlation (*R*^2^ of 72.8%) between AR37 presence in seed, and viability in seedlings. Nevertheless, the correlation was not perfect (regression slope 0.867), as would be predicted if all infected seed gave rise to infected seedlings. We also checked for potential relationships between the seedling emergence rate and % viable endophyte infection rates for maternal plants using simple linear regression analyses, but found poor correlation between these variables (*R*^2^ values ranged from 0 to 2.2% among populations; Supplementary Figure S3).

**FIGURE 7:**
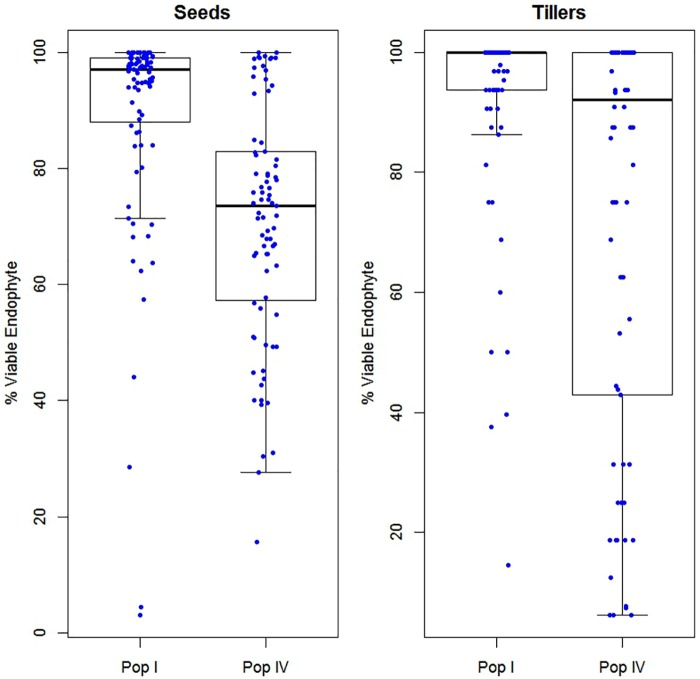
Boxplots showing the distributions of % viable endophyte transmission into seedlings in 2013 (left), and tiller infection levels of parent plants in 2016 (right) in Populations I and IV. Parent plants were grown at the Grasslands Research Centre.

Furthermore, we examined the general distributions of AR37 tiller infection levels and % viable endophyte transmission from maternal parent plants of Populations I and IV (Figure 7). Given these sets of observations were made several years apart, these data have not been analyzed statistically as the stability of these two variables over the time period is unknown. However, in Population I, the majority of parent plants had endophyte transmission rates near 100%, and detection of the presence of endophyte within their tillers showed a similar distribution (Figure 7). By contrast, the tiller infection rates of the Population IV plants had a very broad distribution, which was consistent with the distribution of endophyte transmission rates (Figure 7).

### The Relationship Between AR37 Seed Transmission, and Hyphal Distribution and Concentration in the Tiller Crown

**FIGURE 8:**
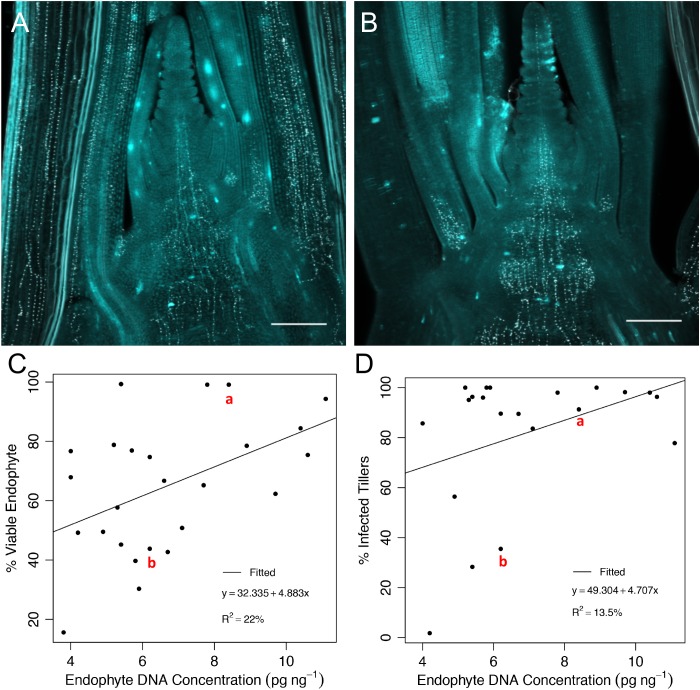
Visualization of endophyte hyphae and hyphal biomass estimates in tiller crown tissues. Confocal microscopy of longitudinal views through the center of tillers showing hyphae in tiller tissues of a representative **(A)** high-transmitting parental genotype, 107 and **(B)** low-transmission genotype, 13 of Population IV. The white dots are labeled septa separating adjacent hyphal compartments (WGA-488, false color white), and scale bar represents 0.2 mm. Scatterplots of the % viable endophyte transmission rates (based on seed harvested at the Grasslands Research Centre in 2013) **(C)** and % tiller infection rates taken in 2016 **(D)**, relative to endophyte DNA concentration in the shoot apex tissues for a subset of 24 parent plant genotypes from Population IV. Data points for the genotypes shown in panels **(A,B)** are labeled in red.

Given that Populations I and IV differed with respect to both the range of % viable endophyte transmission into seed, and the % infected tillers on the maternal plants, we next elected to explore potential relationships between AR37 hyphal distribution and concentration in the TCs with seed transmission. Preliminary observations of AR37 hyphal distribution in TCs of three high (genotype ID 11, 103, and 107; 75.4, 99.3, and 99.1% transmission, respectively) and three low (genotype ID 13, 79, and 83; 43.8, 45.2, and 49.2%) transmitting parent genotypes from Population IV were made using confocal microscopy (Figure 8). The images suggested that the high transmission genotypes generally appeared to have more widespread endophyte distribution and abundance in TCs, as compared to the low transmission genotypes, which generally appeared to have lower endophyte abundance. In particular, the distribution patterns and abundance of AR37 in the stem and shoot apical meristem regions of all samples appeared similar; however, infection of leaves appeared to differ among genotypes, ranging from noticeably reduced (or absent) hyphae in leaf tissue of low transmitting maternal genotypes, to high hyphal density in the leaves of the high-transmitting genotypes. However, genotypes 11 (high seed transmission) and 13 (low seed transmission), both showed intermediate hyphal densities in the leaf tissue.

Based upon these initial observations, we hypothesized that the fungal biomass in the TC may be a determinant of endophyte seed transmission. Therefore, we quantified AR37 biomass, using qPCR, in the TC regions of 25 genotypes from Population IV with a broad range of transmission values, including the genotypes analyzed by confocal microscopy above. Regression analysis between fungal biomass and endophyte transmission (Figure 8) revealed a small, but positive relationship (*y* = 4.883*x* + 32.335, *R*^2^ = 0.22) between endophyte biomass and endophyte transmission. The model fit for the regression equation assessed through *F* statistics was significant (*P* = 0.0195). We also examined the relationship between fungal biomass in the TC and tiller infection rate (Figure 8), but found little correlation (*y* = 4.707*x* + 49.304, *R*^2^ = 0.14).

## Discussion

In nature, the vertical transmission of *Epichloë* hyphae from the mature host grass plant into the seed is the primary mechanism through which the asexual forms of these fungi are disseminated. Since *L. perenne* is a facultative out-crossing species, the endophyte must enter and survive in the embryo of a seed with a different genetic background to that of the colonized maternal parent plant. Therefore, for vertical transmission to be highly efficient, the endophyte must be capable of interacting compatibly with a new host genotype in every seed. However, vertical transmission of *Epichloë* in natural environments is rarely perfect (Gundel et al., 2009), and few studies have attempted to deconvolute the potential impacts of host genetics and environment on this process.

*Epichloë festucae* var. *lolii* strain AR37 was originally isolated from *L. perenne*, and was artificially inoculated into the genotypes from which the pre-breeding populations in this study were derived. Artificial transfer of endophytes between plant genotypes within a species usually results in compatible long lasting symbioses in a proportion of the population, though the degree of compatibility is variable and depends on the endophyte strain and host variety (W. Simpson, pers. comm.). Conversely, transfer of isolates between host species can result in incompatibility which is manifested through a number of aberrant plant and endophyte phenotypes such as increased tillering, reduced plant stature, hyphal or host death, and reduced vertical transmission into seed (Koga et al., 1993; Christensen et al., 1997).

### Host Genotype Influence

In this study, we examined the vertical transmission of *E. festucae* var. *lolii* strain AR37 into approximately 60,000 seedling progeny from over 500 plant genotypes from five independent *L. perenne* populations that were not closely related to the host genotype that AR37 was originally isolated from. We provide evidence from several sets of observations that together demonstrate significant host genetic determinism for endophyte seed transmission. First, GBS to identify genome-wide SNP markers from maternal parent plants, and analyses of these with AR37 vertical transmission frequencies, has enabled us to develop a genomic prediction model to estimate GEBVs (predicted phenotype value) with a relatively high estimated PA of *r* = 0.54. Second, vertical transmission rates for AR37 in vegetatively cloned genotypes from two *L. perenne* populations in the North and South Island of New Zealand showed that, in addition to environmental variables, host genetic variation also has a major bearing on AR37 seed transmission outcomes. Third, in Population III, which had a defined structure comprised of genetically related individuals from 40 half-sibling families, the variation in AR37 seed transmission frequencies among families is significant. Fourth, in ryegrass Populations I–III, that had previously undergone one cycle of selection for improved AR37 transmission, the majority (>70%) of genotypes transmitted viable endophyte into >90% of the progeny seedlings. This is in contrast to the populations that had not undergone selection (IV and V), where only ∼20% of genotypes transmitted viable endophyte at a similar level (Figure 2). Together, these lines of evidence indicate that the genotype of the plant on which the seed formed has a significant impact on the efficiency of AR37 transmission into the progeny.

The development of host GS models for endophyte transmission has not previously been undertaken, and our analyses indicate that GS for AR37 transmission is achievable. GS could therefore significantly improve breeding efficiency for this endophyte transmission by reducing the length of the selection cycle, or by enabling the breeder to better exploit the genetic variation that exists within breeding families (Faville et al., 2018). The relatively high estimated PA (*r* = 0.54) is in accordance with the high degree of genetic determinism for this trait, estimated in a separate experiment for two of the populations (I and IV) as repeatability, *R_c_*, of 0.82–0.92. Plots of GEBV vs. % viable endophyte transmission (BLUP-adjusted means) for the full dataset, as well as a sub-setted dataset (created to have phenotypic bins with equal numbers of genotypes), yielded identical *r* values of 0.55, indicating it unlikely that there was an artefactual influence of skewedness in the phenotypic dataset, on the overall PA estimate. As with an earlier investigation of GS potential for agronomic traits in this training set of five populations (Faville et al., 2018), there was little variation in PA among the range of statistical approaches tested. In that study, it was also demonstrated that application of GS using prediction models with only half the PA of that observed here, could almost double the rate of genetic gain per selection cycle. The degree to which this holds for other host–endophyte symbiota, however, requires further investigation.

Evidence for strong host genetic effects on vertical AR37 transmission is consistent with observations by Saikkonen et al. (2010), who similarly reported variability in vertical transmission of *E. festucae* in the seed from different *Festuca rubra* genotypes. *Festuca rubra* plants artificially infected with *E. festucae* had lower hyphal biomass compared to naturally infected genotypes, and produced more endophyte-free tillers, suggesting that newly created associations were less stable than their naturally infected counterparts. It was concluded that grass genotypes that conferred genetic mismatches between host–endophyte combinations constrain compatible genetic combinations, and can result in reduced vertical transmission rates to seedlings (Saikkonen et al., 2010). A smaller study of endophyte transmission from plant to seed in a number of genotypes of tall fescue infected with *E. coenophiala* also surmised that plant genotype is a key controlling factor (Noh and Ju, 2012). Gundel et al. (2010) predicted that moderate genetic distances between host parents may increase the transmission efficiency of *Epichloë*, but this trend is unlikely to persist when the relative genetic distances between parents are too high (e.g., interspecific) due to out-breeding depression. However, the impact of host genetic background on endophyte vertical transmission was deemed small for *L. multiflorum* infected with *N. occultans* (Gundel et al., 2012). In the *L. multiflorum* study, natural associations of endophyte were examined, and the range of host genetic diversity examined was narrower than in the present study. These factors may have contributed considerably to the differences in host genetics on transmission observed between the two studies.

### Influence of Environment

Environmental factors can alter endophyte transmission efficiency, where their impact may vary depending on the factor and its influence during different stages of the transmission cycle (Figure 1). We observed that the vertical transmission rates of AR37 were higher in LINC as compared to their clonal counterparts grown in GRLDS (Figure 5), and environment was predicted to contribute to the variation in transmission rate observed. This is despite the parent plants for both treatments having been vernalized in GRLDS, and hence, primary reproductive primordia were likely already in place prior to growth in the contrasting environments. Only a small genotype-by-environment interaction was estimated, indicating that genotypes did not re-rank considerably when compared between the two environments. In the LINC and GLRDS environments, both climate and growing media differed, thus it is not possible to discern which factor(s) made the greatest contribution to the differences in seed transmission observed. However, most notably, precipitation was much higher in GRLDS, and temperature lower in LINC, during the September to November period, which is when reproductive development takes place for perennial ryegrass in New Zealand (Lee et al., 2012). Other differences in conditions at each site included the soil/growth medium, which may influence nutrient availability and other biotic and abiotic factors, which were not measured in this study. Few studies to date have systematically investigated the effects of environmental factors on the vertical transmission of endophyte from parent to seeds under controlled conditions. Thus, future studies that examine the impact of factors in isolation, and in combination, will enable identification of environmental conditions that result in higher transmission rates and provide insights into the mechanisms by which these operate.

### Stage of Host–Endophyte Development

It is generally recognized that there are a number of stages in the grass–endophyte life cycle (Figure 1) where vertical transmission of endophyte can fail. Although we have not been able to systematically examine each of these life stages within this study, we have made a number of observations that contribute to our understanding of the influences of these stages on the transmission of *E. festucae* var. *lolii* AR37 within a broad range of *L. perenne* genotypes.

The life-cycle of the host plant may be broadly separated into pre-zygotic (seedling to inflorescence) and post-zygotic (inflorescence to seedling) phases (Gundel et al., 2011b). It has been proposed that, especially during pre-zygotic bud differentiation and anthesis, environment and host genetic influences may have the greatest impact on vertical transmission (Gundel et al., 2011b). In the post-zygotic period, failure of the endophyte to colonize the developing or mature embryo within the seed, or failure of the endophyte within the embryo to colonize key meristematic tissues in the seedling will impair endophyte infection status. We observed a high correlation between endophyte presence in seed, and viable endophyte within the seedlings from the same seed pools of a range of parental genotypes (Figure 6) which suggests that transmission of AR37 from recently harvested seed to seedling is generally an efficient process within these ryegrass populations, and not a major impediment in the vertical transmission of endophyte from parent to progeny. This may not be the case for all host–endophyte associations, however. A recent study of the vertical transmission of an artificial symbiosis between *L. perenne* with the *L. arundinaceum* (tall fescue) endophyte, *Epichloë* sp. FaTG-3 strain AR501 showed that environmental factors can have a large impact on endophyte viability at the seed to young seedling stage (Freitas, 2017). In this study, seedlings were assigned to different temperature treatments 2 weeks after sowing, and endophyte status checked 3 weeks thereafter. The infection frequency of seedlings was significantly higher for those raised in a cool temperature regime (day/night 12/6°C) compared to those raised in a warm regime (day/night 25/16°C) (Freitas, 2017). Thus growth temperature post-germination can have a large impact on overall vertical transmission, though the degree to which this occurs in other grass endophyte symbioses, particularly for endophytes within their native host species, requires further investigation.

For endophyte-infected seed, retention of endophyte viability is optimal under both low temperature and humidity conditions, where loss of viability can be significant under elevated temperature and humidity (Rolston et al., 1986; Hume et al., 2013). Despite optimal storage conditions for endophyte viability being used in this study, it is often observed that the proportion of endophyte infected seedlings is lower than that of seeds, the degree of this difference appears to be host genotype and endophyte strain specific (unpubl. obs.). The causes of this decrease remain unclear however, and require further investigation. In our study, the presence of AR37 DNA within individual seeds was determined, but not the location of endophyte hyphae within the seed, or physiological or metabolic status differences between seeds. Thus we cannot discern whether the embryo or infection layer was colonized or that the endophyte was viable in all endophyte-infected seeds. Moreover, the efficiency of hyphal colonization of the shoot apex of the germinated seedling is not known. This information will enable further identification of critical aspects of development that may contribute to the decreased incidence of endophyte within seedlings compared to seed.

In our study, the emergence rates among the five ryegrass populations during the sowing of seed to assess endophyte transmission did not strongly correlate with endophyte infection. However, in the study of Clay (1987), perennial ryegrass and tall fescue seeds from infected plants were shown to have a higher rate of germination than seeds from un-infected plants. The superior fitness of infected seedlings may be due to benefits conferred by endophyte bioactive compounds such as herbivory deterrence, antimicrobial activities, or modulation of plant physiology. However, failure of seed to germinate may be due to empty seed, dead seed, abnormally developed seed, or as a result of host developmental issues. Conceivably, such issues would also disrupt the finely synchronized and coordinated development of host and endophyte for colonization of floret, ovule, seed, and embryo required for successful vertical transmission of viable endophyte to seedling progeny.

Pre-zygotic influences appear to be more critical to vertical transmission among the host–endophyte associations of this study. Failure of the endophyte to colonize axillary buds and floral primordia are two events which may significantly impact vertical transmission. Population IV, a population derived from a cross between a late flowering New Zealand cultivar and a European cultivar, displayed a particularly broad range of vertical transmission rates. We investigated Population IV further after unexpectedly finding that a large proportion (∼27%) of parent plants had become endophyte-free over the course of 1 year of vegetative growth and propagation. Loss of endophyte infection within AR37-infected ryegrass is not uncommon (unpubl. obs.); however, the underlying mechanism(s) are not understood. Nonetheless, the mechanisms are likely to involve incompatibilities between host and endophyte that may kill the endophyte or prevent efficient colonization of axillary buds, resulting in endophyte-free daughter tillers (and all subsequent daughter tillers). Related to this, ramet selection for clonal propagation may have accelerated loss of endophyte if the subset of tillers used for propagation were from an endophyte-free section of the plant. Therefore, the efficiency of axillary bud colonization is presumed to play a large role in the rate at which endophyte-free plants were generated *via* vegetative propagation. To further explore this, we examined the tiller infection levels of parent plants and found that the general trend of a broad range of seed transmission among both Population IV genotypes was reflected by the broad distribution of tiller infection rates (Figure 7).

We sought to further investigate factors that may lead to imperfect axillary bud infection. It has been suggested that higher fungal biomass in host tissues may result in more effective vertical transmission; however, evidence for a direct linkage is presently lacking (Gundel et al., 2011b). Endophyte biomass in the TC regions of a range of parent plant genotypes from Population IV showed no or very little correlation with tiller infection level, and a small, but positive relationship with seed transmission (Figure 8), respectively. Although few genotypes were examined, these data suggest that the ability of the endophyte to colonize new axillary buds may be relatively independent of its concentration in the TC. However, the localization of hyphae in close proximity to developing primordia is likely to be vital for successful transmission of endophyte to every tiller, and detailed determination of hyphal location in the shoot apex is recommended as a focus of future investigations, in addition to examining biomass across a broader range of host genotypes. Environment has been shown to influence tiller infection rates; however, where in tall fescue, the tiller infection frequency by *E. coenophiala* was lower in cold seasons compared to warmer ones (Ju et al., 2006).

Our observations of AR37 indicate that bottlenecks in vertical transmission occurred during both vegetative and sexual stages of the host life cycle, first through failure to infect clonal daughter tillers, and later during colonization of inflorescences, seed or seedlings. The molecular basis for these bottlenecks is not yet understood, but may relate to incompatibility between host and endophyte. Transcriptomic analyses of *L. perenne* responses to a *L. pe*renne endophyte (Lp19) (Schmid et al., 2017) as compared to an endophyte from a more distantly related host (Fl1 from *Festuca trachyphylla*) (Dupont et al., 2015) indicate large scale changes in host gene expression in response to both endophytes, but little commonality in response between the two. This suggests that *L. perenne* reactions to Lp19 and Fl1 endophyte associations are not the same (Schmid et al., 2017). However, there was no evidence for upregulation of host defense response genes in the Fl1 association (Dupont et al., 2015), so the implications of host gene expression changes on potential incompatibilities between the organisms remain unresolved. While there is little known about the host determinants of incompatibility, the targeted deletion of *E. festucae* genes indicated that disruption of processes involving reactive oxygen species synthesis, iron regulation, cell signaling and others, may profoundly affect the phenotypes of the host and endophyte, often through unregulated hyphal growth (Tanaka et al., 2006; Johnson et al., 2013b; Voisey et al., 2016). Such manipulations are useful in understanding fundamental process underlying the association, but cannot be used to infer how different *L. perenne* genotypes may respond to infection by a presumed homogenous isolate. We intend to address this question using transcriptomic analyses.

Although we did not directly examine endophyte colonization of reproductive tissues in this study, previous studies have reported variation in endophyte infection frequency based on floret position within spikelets. Floret and seed infection rates were generally higher at the terminal end of flowering spikelets as compared to basal positions, for both tall fescue (Noh and Ju, 2012) and perennial ryegrass (Wang et al., 2018). Inflorescence development follows a detailed sequence of events that involve opposing vegetative and reproductive gradients along the developing spikelet axis (Evans and Grover, 1940). Wang et al. suggest that the endophyte may grow more slowly in the basal positions, and thus are more likely to miss the fine window of opportunity to colonize the developing seed and embryo. Moreover, environmental factors such as nutrient type (Wang et al., 2018) and water availability (Davitt et al., 2011) have been shown to influence seed transmission rates and inflorescence number.

### Summary

From the GBS analysis of over 500 genotypes of *L. perenne* infected with *E. festucae* var. *lolii* strain AR37, we have demonstrated that the vertical transmission of AR37 to progeny *via* the seed is heavily influenced by host genetic factors. We have also shown transmission frequency is greater in the cooler, drier climate of Lincoln compared to Grasslands, though the specific factors underlying this increase are not known. Genotype-by-environment interactions are also operating, but these are relatively small compared to those of either genotype or environment alone. Within the endophyte–host developmental cycle, low vertical transmission is associated with low tiller infection frequencies for one of the breeding populations examined. It is thus assumed that the efficiency of axillary bud colonization from the shoot apex, required for tiller infection, is poor in many of these associations. Our future studies will focus on using the *L. perenne* SNP information to identify genes and biomarkers that are involved in key developmental and physiological processes that influence vertical transmission, with the aim of enhancing rates for agriculturally important grass–endophyte associations.

## Data Availability

The datasets for this manuscript are not publicly available because they belong to a third party. Requests to access the datasets should be directed to MF, marty.faville@agresearch.co.nz.

## Author Contributions

CV, MG, JK, MF, HE, RJ, and LJ conceived and designed the study. MG, DH, MR, WZ, and NF conducted experimental work. MG, JK, SG, MF, WZ, RJ, CM, and CV analyzed and interpreted the data. CM, MG, MF, WZ, NF, and CV contributed to the writing and editing of the manuscript.

## Conflict of Interest Statement

The authors declare that the research was conducted at AgResearch, a New Zealand Crown Research Institute. Neither the funders (New Zealand Ministry of Business, Innovation and Employment) nor the donor of the plant material (Grasslands Innovation, or its shareholders, Grasslanz Technology Limited and PGG Wrightson Seeds), had a role in study design, data collection and analysis, or preparation of the manuscript.

## Footnotes

1 https://cliflo.niwa.co.nz/
