# Supplementary material for: Seed Transmission of Epichloë Endophytes in Lolium perenne Is Heavily Influenced by Host Genetics
Source: Front Plant Sci. 2018 Nov 13;9:1580. doi: 10.3389/fpls.2018.01580 (PMC6242978; doi:10.3389/fpls.2018.01580)
Supplement: Supplementary file 1 [file Data_Sheet_1.pdf]

Supplementary Material

Seed transmission of *Epichloë* endophytes in *Lolium perenne* is heavily influenced by host genetics

Milan Gagic[1], Marty J. Faville[1], Wei Zhang[1], Natasha T. Forester[1], M. Philip Rolston[2], Richard D. Johnson[1], Siva Ganesh[1], John P. Koolaard[1], H. Sydney Easton[1], Debbie Hudson[1], Linda J. Johnson[1], Christina D. Moon[1] and Christine R. Voisey[1*]

[1]AgResearch, Grasslands Research Centre, Palmerston North, New Zealand

[2]The Foundation for Arable Research, Templeton, Christchurch, New Zealand

***Correspondence:** Christine R. Voisey, christine.voisey@agresearch.co.nz

Supplementary Figure 1. Total rainfall (right) and mean, maximum and minimum temperature (left) each month at the Grasslands (GRLDS) and Lincoln (LINC) Research Centres during the 12 month periods that the seed transmission trials were conducted.

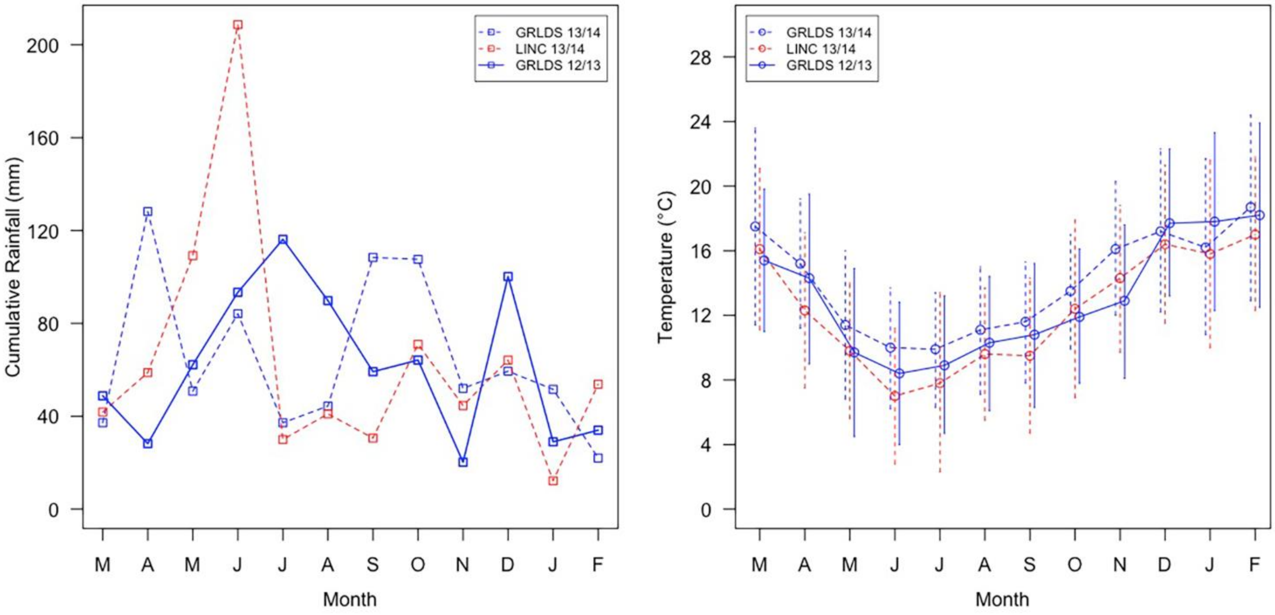

Supplementary Figure 2. Genomic-estimated breeding values (GEBVs) plotted against observed phenotypic values (BLUP-adjusted means) for endophyte transmission % in the ryegrass training set. Panel A, full dataset, B, reduced dataset of n = 81, based on random selection of individuals within each of the intervals 0 – 20 (n = 5), 21 – 40 (n = 16), 41 – 60 (n = 20), 61 – 80 (n = 20) and 81 – 100 (n = 20). GEBVs were determined using the KGD statistical method.

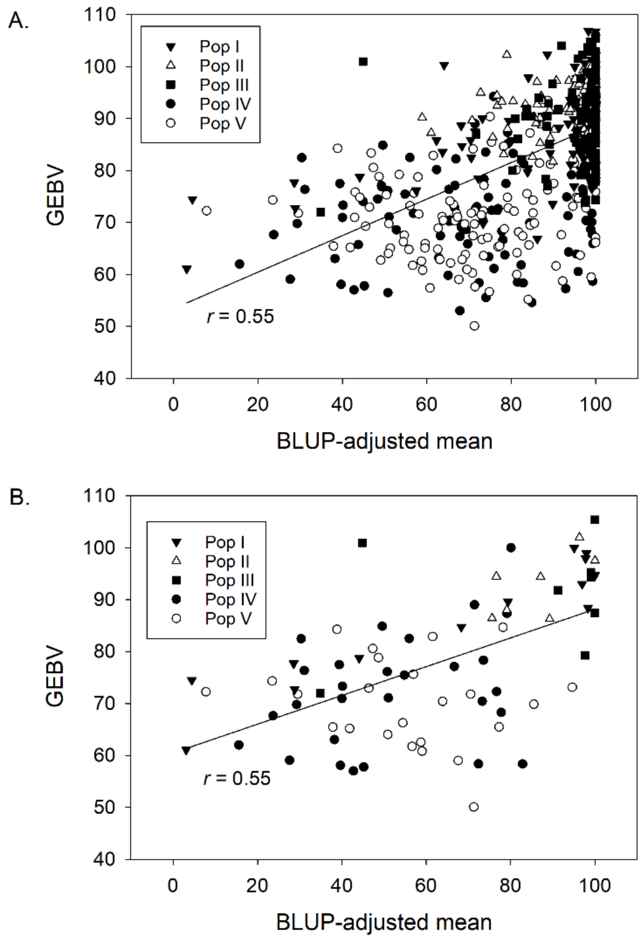

Supplementary Figure 3. Relationships between % viable endophyte transmission and seedling emergence rates for Populations I-V (panels A to E), and all populations together (F). Data has been arcsin transformed and simple linear regression analysis is shown.

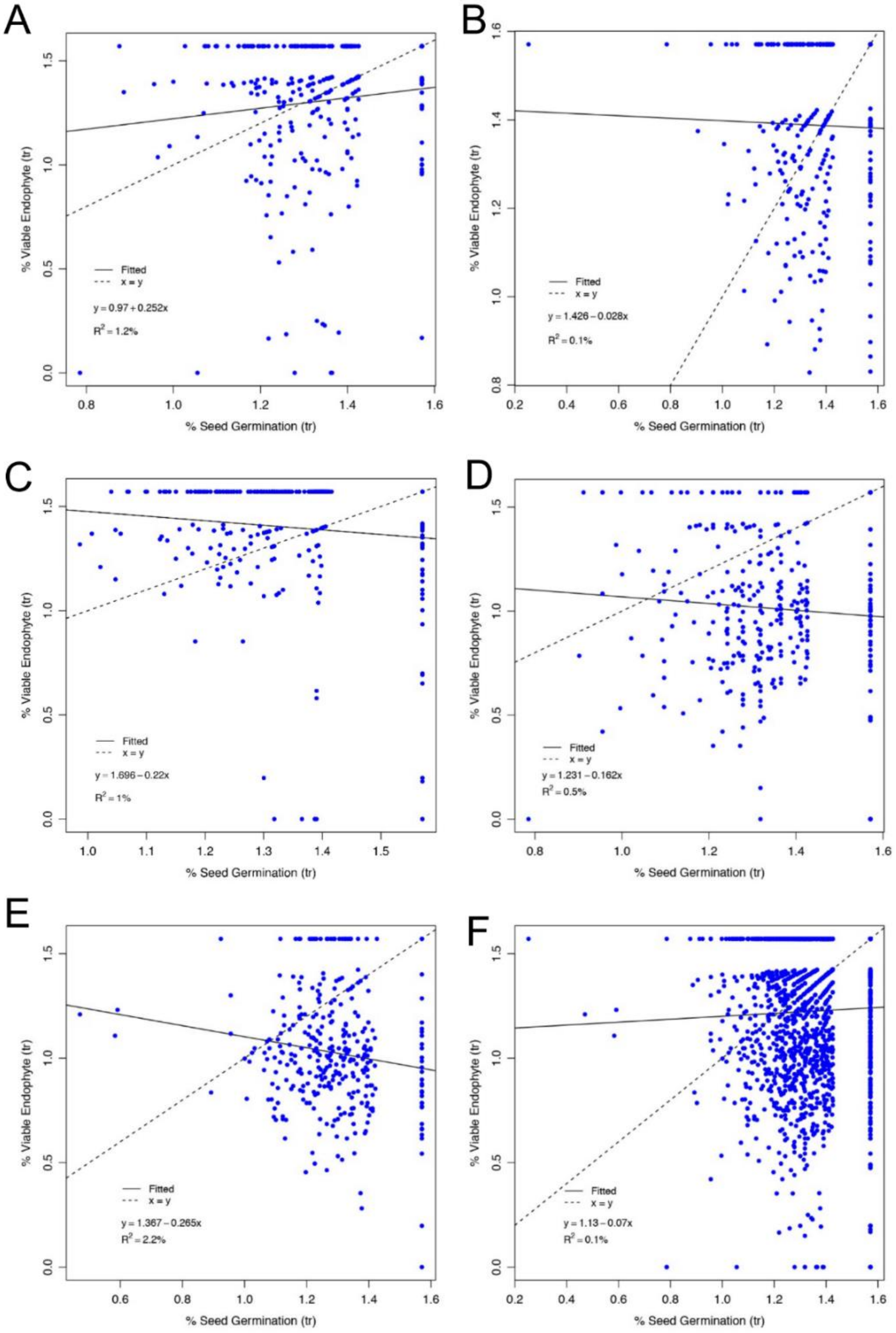

Supplementary Table 1. Genetic structure of Population III showing the relationships between maternal parent genotypes as half-sibling families.

Family	Parent genotype IDs		
1	111	112	
2	121	122	123
3	131	132	
4	141	142	143
5	151	152	
6	211	212	213
7	221	222	
8	231	232	233
9	242		
10	251	252	253
11	311	312	
12	321	322	323
13	331		
14	341	342	343
15	351	352	
16	411	412	413
17	421	422	
18	431	432	
19	441	442	
20	451	452	453
21	511	512	
22	521		
23	531	532	
24	541	543	
25	551	552	
26	612	613	
27	621	622	
28	631	632	
29	641	642	
30	651	652	
31	711	712	
32	721	722	723
33	731		
34	741	742	743
35	751	752	
36	811	812	
37	821		

38	831	832	833
39	841	842	
40	851	852	853
41	911	912	
42	921	922	923
43	931	932	
44	941	942	943
45	951	952	
46	1011	1012	
47	1021	1022	
48	1031	1032	1033
49	1041	1042	1051
50	1052	1053	

Supplementary Table 2. ANOVA for % viable endophyte transmission rates[1] within Population III.

Source of variation	df	MS	F	*P*-value
HS families	49	0.491	2.044	0.021
Lines in HS families	63	0.240	12.522	1.52E-45
Error	224	0.019		
Total	36			

[1] Endophyte transmission rates are from seed harvested in 2013. Data has been arcsin transformed.

Supplementary Table 3. Observed % viable endophyte transmission and GEBV data

Population	Genotype ID	% viable endophyte transmission (BLUP)	GEBV[1] mean	GEBV SE[2]
I	1	94.79	89.96	2.85
I	2	28.57	77.71	2.07
I	3	80.20	85.92	2.23
I	4	84.07	97.91	1.79
I	5	63.73	83.55	2.42
I	6	44.07	78.75	1.07
I	8	100.00	78.88	0.65
I	9	100.00	106.79	0.79
I	10	93.51	89.03	1.64
I	11	99.02	85.85	0.92
I	13	91.34	88.28	1.41
I	14	97.44	100.62	1.53
I	15	100.00	100.40	1.02
I	17	95.65	84.83	0.95
I	18	94.85	85.83	2.45
I	19	98.95	82.52	1.50
I	20	96.62	90.59	2.58
I	21	97.46	84.31	2.59
I	23	100.00	92.98	2.42
I	24	94.69	83.25	1.18
I	25	3.09	61.12	2.42
I	26	99.16	94.25	1.89
I	27	95.06	100.00	1.32
I	28	96.75	91.37	2.19
I	30	99.04	96.92	1.49
I	31	100.00	81.23	1.62
I	32	96.94	93.00	2.76
I	33	97.96	98.93	1.73
I	37	89.83	73.55	3.41
I	38	73.17	90.03	1.71
I	39	97.92	90.89	5.28
I	40	97.35	87.89	1.62
I	43	100.00	94.18	0.87
I	44	93.97	93.56	1.86
I	45	95.45	97.80	4.05
I	47	97.86	88.54	2.17
I	48	99.04	83.67	3.04

I	49	97.14	82.25	3.44
I	51	64.10	100.35	2.49
I	53	100.00	94.69	0.85
I	56	98.96	101.32	1.07
I	57	97.92	83.89	2.25
I	59	100.00	78.13	2.48
I	60	97.39	96.41	1.92
I	62	86.17	66.85	1.96
I	63		87.75	2.11
I	64	70.40	85.89	2.31
I	66	87.36	90.57	1.97
I	67	89.23	83.38	1.11
I	68	98.96	89.32	3.13
I	70	73.33	69.79	2.09
I	71	71.43	87.48	3.76
I	72	72.19	78.50	0.85
I	73	97.70	85.53	1.24
I	76	100.00	99.40	1.75
I	77	96.40	80.20	2.39
I	78	83.96	80.42	1.36
I	81	70.53	82.50	2.55
I	82	98.08	80.46	3.01
I	83	94.21	92.56	2.81
I	84	94.68	86.44	2.22
I	85	97.78	97.93	1.34
I	86	97.44	90.59	1.51
I	88	99.41	76.98	5.54
I	89	98.33	92.92	1.03
I	90	83.87	79.71	1.51
I	92	4.42	74.51	2.16
I	93	96.59	83.02	2.80
I	95	95.45	92.39	1.48
I	96	99.32	81.91	2.13
I	97	62.28	85.81	1.14
I	98	98.29	106.87	1.68
I	99	97.66	88.02	2.20
I	100	68.32	84.74	3.80
I	101	57.48	76.16	0.70
I	102	97.09	87.60	1.87
I	103	98.33	88.41	1.46
I	105	98.48	89.56	1.15
I	108	97.52	96.58	3.03

I	109	99.30	102.78	1.99
I	110	100.00	105.57	1.29
I	112	96.25	83.78	2.28
I	113	100.00	77.43	2.04
I	114	100.00	94.32	1.99
I	115	99.31	81.85	2.77
I	116	100.00	102.25	1.75
I	119	99.12	76.54	7.58
I	121	100.00	92.11	1.96
I	123	86.36	89.60	1.15
I	124	79.38	89.62	3.28
I	125	100.00	92.46	1.11
I	126	28.79	72.75	1.99
I	128	88.54	102.34	1.11
I	129	94.06	81.14	0.57
I	130	94.87	76.96	2.41
I	131	100.00	80.98	2.43
I	132	68.14	88.64	1.08
II	1	96.30	101.97	1.22
II	2	97.14	86.98	0.99
II	4	100.00	96.46	0.74
II	5	93.67	91.26	2.53
II	6	100.00	103.06	1.56
II	7	96.04	96.65	0.87
II	8	97.06	91.36	1.25
II	9	98.00	87.03	1.73
II	10	87.21	88.68	1.76
II	11	97.67	90.14	1.22
II	12	95.28	88.11	1.59
II	13	100.00	91.88	1.50
II	14	89.25	91.44	1.10
II	15	75.61	86.39	1.96
II	16	89.87	81.72	0.75
II	17	100.00	95.29	1.52
II	18	98.78	91.86	0.90
II	19	100.00	96.52	1.52
II	20	100.00	97.47	0.93
II	21	58.88	90.19	0.84
II	22	86.79	90.08	1.91
II	23	98.77	88.92	1.06
II	24	86.84	85.28	0.76

II	25	96.39	96.81	1.36
II	26	97.94	93.41	1.69
II	27	97.06	99.29	1.28
II	28	87.10	94.34	0.66
II	29	100.00	87.76	1.17
II	30	100.00	93.57	0.73
II	31	100.00	93.38	0.78
II	32	78.21	83.12	1.61
II	33	95.12	97.57	1.40
II	34	87.50	93.44	1.08
II	35	100.00	92.95	1.79
II	37	94.67	94.55	1.23
II	38	93.68	97.28	1.17
II	39	95.12	96.33	1.53
II	40	100.00	91.48	0.66
II	43	85.00	82.61	1.03
II	44	95.15	97.64	1.13
II	45	98.85	90.37	0.65
II	46	100.00	97.00	2.27
II	48	98.23	93.11	0.29
II	49	98.68	91.16	1.93
II	50	99.17	95.47	1.09
II	51	100.00	94.68	1.23
II	52	96.47	92.22	0.53
II	53	98.70	92.59	1.10
II	54	97.26	87.89	1.62
II	55	100.00	93.76	2.25
II	56	88.66	88.25	0.45
II	57	96.94	98.31	0.99
II	58	79.09	87.97	1.10
II	59	72.73	94.99	1.43
II	60	70.73	89.65	1.15
II	61	99.06	90.25	2.59
II	62	77.78	93.25	1.64
II	63	87.50	93.37	0.42
II	64	99.05	94.07	0.92
II	65	98.91	87.53	1.66
II	66	100.00	94.92	1.54
II	67	97.50	95.31	0.24
II	68	97.22	95.38	1.23
II	69		102.23	0.93
II	70	86.11	97.05	1.09

II	71	83.33	92.71	0.74
II	72	61.02	87.25	1.05
II	73	100.00	95.24	0.94
II	74	99.12	93.23	1.58
II	75	85.45	90.23	0.71
II	76	97.06	91.40	0.83
II	77	97.80	96.27	1.31
II	78	98.68	90.25	0.77
II	79	98.21	89.08	0.66
II	80	98.91	91.76	1.88
II	81	96.00	93.68	0.58
II	82	97.83	90.19	0.61
II	83	99.22	94.48	1.79
II	84	99.03	93.16	1.29
II	85	99.04	100.12	0.67
II	86	88.97	91.38	1.39
II	87	99.29	89.66	1.83
II	88	90.68	97.30	0.61
II	89	97.94	94.30	0.74
II	90	98.84	93.93	2.25
II	92	93.20	91.91	0.84
II	93	100.00	98.07	0.63
II	95	89.09	91.62	1.48
II	96	98.11	97.32	0.41
II	97	94.07	91.82	0.81
II	98	96.67	93.13	1.29
II	99	100.00	94.38	0.96
II	100	76.67	92.46	2.01
II	101	87.37	92.69	1.06
II	102	98.75	84.44	1.65
II	103	76.61	94.43	0.99
II	104	100.00	94.61	1.40
II	105	85.88	89.09	2.51
II	108	99.19	98.47	1.39
II	109	99.01	94.85	1.05
II	110	97.78	84.26	2.14
II	111	94.92	96.20	0.57
II	112	100.00	90.26	0.98
II	113	96.81	96.09	1.63
II	114	80.43	93.36	1.41
II	115	100.00	93.61	0.92

II	116	90.11	87.82	0.92
II	117	90.91	91.38	1.26
II	119	89.25	86.25	1.14
II	120	86.17	92.76	1.59
III	111		84.15	2.38
III	112	80.23	79.97	5.31
III	113		91.75	3.67
III	121	95.65	87.73	4.50
III	122	44.83	100.99	1.51
III	131	96.85	102.28	1.21
III	132	100.00	94.63	2.55
III	141	82.47	82.19	2.21
III	142	99.09	89.35	1.22
III	143	100.00	86.40	1.46
III	151	100.00	77.93	4.91
III	152	100.00	83.38	3.23
III	211	100.00	91.79	3.60
III	212	98.89	83.61	1.89
III	213	83.70	91.78	1.41
III	221	100.00	78.88	2.51
III	222	100.00	91.78	1.20
III	231	97.50	73.81	1.68
III	232	95.24	93.19	1.29
III	233	96.81	90.69	2.74
III	242	98.95	80.82	3.17
III	251	100.00	74.37	2.16
III	252		87.91	1.83
III	253	34.83	71.99	0.82
III	311	100.00	101.53	2.63
III	312	81.00	89.99	2.30
III	321	97.67	92.59	1.54
III	322	98.61	91.19	2.95
III	323	99.12	94.31	2.93
III	331	96.51	97.70	1.14
III	341	98.99	83.79	2.74
III	342	96.47	91.37	3.46
III	343	95.45	85.42	3.13
III	351	94.44	95.07	1.33
III	352	97.53	86.15	2.08
III	411	91.95	104.05	2.23
III	412	98.98	94.32	1.60
III	413	100.00	90.32	2.28

III	421	91.18	91.77	1.95
III	422	100.00	97.33	1.94
III	431	88.89	92.89	1.59
III	432	97.75	93.86	2.39
III	441	100.00	92.62	3.36
III	442	100.00	88.04	2.31
III	451	89.06	85.04	6.23
III	452	98.81	104.18	1.77
III	453	81.58	86.10	1.32
III	511	97.87	98.92	1.77
III	512	100.00	94.03	3.18
III	521	95.08	86.30	3.39
III	531	83.33	90.44	2.23
III	532	88.64	77.02	3.64
III	541	88.33	88.60	1.48
III	543	95.83	101.54	0.89
III	551	99.17	85.03	2.42
III	552	100.00	101.32	1.07
III	612	84.78	90.42	3.79
III	613	88.51	90.44	2.16
III	621	100.00	102.90	2.86
III	622	96.74	84.23	5.39
III	631	96.43	79.12	2.27
III	632	100.00	95.50	3.92
III	641	100.00	87.39	3.64
III	642	100.00	90.20	1.69
III	651	100.00	105.80	2.44
III	652	98.80	104.78	1.35
III	711	100.00	105.38	2.08
III	712	98.89	86.46	1.73
III	721	99.03	94.59	1.53
III	722	100.00	90.98	2.51
III	723	100.00	98.32	1.89
III	731	89.74	96.69	0.83
III	741	99.09	104.02	1.03
III	742	100.00	91.88	1.56
III	743	99.05	95.22	0.55
III	751	97.67	74.39	6.20
III	752		92.06	1.41
III	811	86.52	94.03	2.87
III	812	98.55	84.77	0.93

III	821	97.10	75.64	1.20
III	831	100.00	98.07	2.55
III	832	88.16	78.32	1.37
III	833	94.37	93.89	1.67
III	841	99.09	97.51	1.11
III	842	99.00	96.31	2.51
III	852	96.47	84.40	2.43
III	853	100.00	98.70	2.00
III	911	97.89	91.05	2.56
III	912	98.33	90.53	2.62
III	921	100.00	79.90	1.48
III	922	100.00	80.71	2.85
III	923	100.00	86.07	2.35
III	931	71.72	87.01	1.88
III	932	97.59	79.27	2.73
III	941	100.00	81.98	2.96
III	942	100.00	100.55	2.21
III	943	100.00	92.50	1.11
III	951	99.07	93.33	1.72
III	952	100.00	86.59	2.04
III	1011	98.82	102.87	2.34
III	1012	100.00	85.22	1.27
III	1021	100.00	90.85	2.66
III	1022	98.15	103.71	1.66
III	1031	100.00	89.07	2.39
III	1032	100.00	99.04	2.02
III	1033	96.63	89.53	2.31
III	1041	98.57	94.30	0.47
III	1042	98.88	89.89	7.11
III	1051	100.00	99.96	2.10
III	1052	99.00	98.61	1.66
III	1053	100.00	99.48	1.78
IV	2	76.87	72.64	4.57
IV	3	78.03	66.65	4.14
IV	4	30.34	82.46	1.21
IV	5	94.32	79.17	1.71
IV	6	72.39	58.38	1.04
IV	7	79.13	87.35	0.95
IV	8	50.78	56.48	0.97
IV	9	78.52	63.74	3.07
IV	11	75.41	72.33	3.34
IV	12	77.78	68.29	2.54

IV	13	43.75	65.74	3.42
IV	14	68.18	69.10	2.00
IV	15	80.45	83.28	2.82
IV	17	49.55	84.88	1.35
IV	18	66.91	82.23	3.01
IV	19	62.31	80.16	2.41
IV	21	96.91	76.41	2.81
IV	23	98.88	78.40	2.35
IV	24	38.21	63.05	1.26
IV	25	31.07	76.34	3.47
IV	26	39.67	58.08	2.73
IV	29	84.42	69.95	4.27
IV	30	92.91	57.27	2.19
IV	31	62.83	63.20	1.71
IV	32	65.26	70.23	4.65
IV	33	74.62	74.82	3.57
IV	34	84.91	54.58	3.07
IV	35	39.38	77.48	1.84
IV	36	51.05	71.08	1.68
IV	37	68.53	69.65	2.88
IV	39	73.95	83.54	1.53
IV	40	93.46	71.28	3.55
IV	42	71.43	89.01	3.05
IV	43	79.17	85.15	2.71
IV	44	73.30	70.42	2.43
IV	45	66.67	72.75	2.47
IV	46	65.49	70.47	2.70
IV	47	66.67	77.11	4.68
IV	48	63.21	67.44	1.47
IV	49	15.60	61.99	1.03
IV	50	69.33	68.66	3.13
IV	52	49.31	76.85	2.61
IV	53	27.61	59.08	4.74
IV	54	99.37	65.93	3.07
IV	55	48.35	74.51	1.39
IV	56	0.72	72.89	1.98
IV	57	78.76	77.49	3.25
IV	58	50.76	76.12	1.13
IV	59	65.22	76.35	2.60
IV	60	73.56	78.31	5.27
IV	64	52.78	68.57	2.26

IV	65	75.81	94.25	2.45
IV	67	44.86	74.07	0.76
IV	68	74.68	63.40	1.34
IV	69	75.93	61.12	4.84
IV	70	65.03	59.81	4.38
IV	71	56.86	71.77	1.15
IV	72	54.86	75.46	3.42
IV	73	40.13	73.32	3.67
IV	74	55.97	82.51	1.28
IV	75	82.98	81.29	2.43
IV	77	69.71	65.86	1.22
IV	78	23.72	67.66	3.83
IV	79	45.18	57.78	0.86
IV	80	29.27	69.78	2.69
IV	82	93.15	74.90	3.14
IV	83	49.22	77.09	2.63
IV	84	40.00	70.95	6.91
IV	85	82.35	61.82	1.76
IV	86	42.74	57.02	7.38
IV	87	95.88	60.54	2.53
IV	88	93.62	64.31	2.33
IV	89	97.71	70.37	5.42
IV	90	99.31	58.68	3.76
IV	92	98.84	68.72	1.80
IV	95	71.60	72.77	4.51
IV	96	81.51	58.51	1.90
IV	97	99.06	70.07	3.68
IV	98	99.12	77.79	2.18
IV	99	98.91	83.36	2.87
IV	101	100.00	90.20	2.68
IV	102	95.45	63.92	1.08
IV	103	99.34	77.28	2.14
IV	104	67.86	67.29	1.61
IV	107	99.14	71.75	3.28
IV	108	100.00	80.08	2.75
IV	109	76.67	72.29	1.11
IV	110	74.02	55.53	4.75
IV	111	67.83	53.01	4.26
IV	112	99.20	67.71	1.34
IV	114	82.86	58.36	2.65
IV	115	71.83	73.03	3.56
IV	116	97.44	80.92	1.94

V	1	56.96	75.63	2.47
V	2	96.36	63.68	3.40
V	3	76.92	65.30	3.05
V	5	43.01	75.83	1.65
V	6	58.73	62.56	2.68
V	7	84.46	87.21	1.65
V	8	54.65	67.53	1.36
V	9	59.62	65.92	0.89
V	10	81.16	80.10	1.74
V	11	71.43	57.61	2.05
V	12	61.54	82.89	1.47
V	13	67.59	59.01	1.18
V	14	69.14	63.82	1.30
V	15	66.67	70.33	0.98
V	16	41.84	65.18	1.83
V	17	51.35	65.03	2.60
V	18	68.38	62.84	2.34
V	19	70.97	67.43	2.53
V	20	88.66	93.47	5.21
V	21	46.77	83.36	1.28
V	22	81.75	68.17	1.60
V	23	50.91	64.03	0.36
V	24	86.73	81.45	3.00
V	25	94.63	73.12	3.93
V	27	84.52	69.44	1.33
V	28	53.47	79.22	0.85
V	29	29.51	71.78	1.50
V	30	57.89	78.76	1.42
V	31	63.64	71.13	1.60
V	32	91.89	67.63	1.88
V	33	72.92	71.44	3.56
V	34	70.83	60.28	2.89
V	35	79.27	72.76	2.97
V	37	83.75	75.54	0.54
V	38	95.41	74.83	2.62
V	39	46.39	72.94	1.42
V	40	70.53	71.79	1.67
V	43	73.10	66.73	1.27
V	44	92.22	58.73	3.44
V	45	62.70	68.89	3.96
V	46	77.27	65.47	3.12

V	47	59.02	60.81	0.79
V	48	79.12	74.82	0.92
V	49	69.51	60.96	2.92
V	51	67.07	71.71	2.86
V	52	100.00	89.58	1.72
V	54	84.04	55.19	1.24
V	55	70.97	84.15	1.58
V	56	73.53	77.15	1.57
V	57	90.63	61.62	0.72
V	58	85.51	69.87	1.75
V	59	85.19	74.29	1.79
V	60	78.22	84.67	2.60
V	61	95.88	74.66	1.56
V	62	74.42	67.07	2.01
V	63	66.30	73.47	1.90
V	64	58.10	71.62	1.31
V	65	71.29	50.09	2.13
V	66	68.94	69.70	1.75
V	67	81.34	59.97	1.62
V	68	62.60	64.84	2.29
V	69	42.86	70.96	2.28
V	70	74.81	56.66	0.89
V	71	60.75	57.39	5.16
V	72	45.45	69.29	0.51
V	73	55.75	64.80	3.71
V	74	75.00	90.38	1.37
V	75	100.00	75.92	1.46
V	76	51.02	69.77	2.65
V	77	85.19	71.82	0.80
V	78	47.31	80.58	2.64
V	79	7.79	72.26	0.95
V	80	73.47	63.62	2.77
V	81	82.72	67.39	4.65
V	82	84.21	65.65	2.18
V	83	71.30	69.70	2.23
V	84	67.29	71.05	3.01
V	85	48.65	78.82	1.78
V	86	65.63	62.19	1.25
V	87	65.49	74.14	1.56
V	88	63.89	70.39	0.74
V	89	44.07	74.84	2.71
V	90	56.67	61.75	1.05

V	92	88.64	76.26	1.48
V	93	54.46	66.30	2.68
V	95	63.11	62.87	0.96
V	96	37.86	65.46	1.82
V	97	50.38	75.32	0.87
V	98	59.55	65.07	3.16
V	99	80.70	72.18	5.70
V	100	95.00	81.43	1.67
V	101	73.39	72.19	3.82
V	102	90.07	77.50	2.28
V	103	38.82	84.26	2.50
V	104	71.29	72.20	2.01
V	105	81.40	64.18	2.32
V	108	49.06	62.74	2.47
V	109	78.35	68.77	0.67
V	110	100.00	85.55	2.13
V	111	23.47	74.34	2.85
V	112	56.03	73.23	3.78
V	113	98.95	59.50	2.96
V	114	75.34	85.44	2.14
V	115	94.67	82.02	1.07
V	116	89.32	81.21	1.35
V	117	97.70	72.79	2.26
V	119	100.00	67.11	2.75
V	120	100.00	66.16	1.56

[1] GEBV values are derived using the KGD-GBLUP method.

[2] Standard errors of the GEBV values are shown.
